# Factors associated with loss to clinic among HIV patients not yet known to be eligible for antiretroviral therapy (ART) in Mozambique

**DOI:** 10.7448/IAS.16.1.18490

**Published:** 2013-06-10

**Authors:** Rituparna Pati, Maria Lahuerta, Batya Elul, Mie Okamura, Maria Fernanda Alvim, Bruce Schackman, Heejung Bang, Rufino Fernandes, Americo Assan, Josue Lima, Denis Nash, for the Identifying Optimal Models of HIV Care in Mozambique Study Group

**Affiliations:** 1 Spencer Cox Center for Health, St. Luke's and Roosevelt Hospitals, New York, United States; 2 Weill Cornell Medical College, Departments of Medicine and Public Health, New York, United States; 3 ICAP, Columbia University, Mailman School of Public Health, New York, United States;; 4 Columbia University, Mailman School of Public Health, Department of Epidemiology, New York, United States; 5 Weill Cornell Medical College, Department of Public Health, New York, United States; 6 Division of Biostatistics, Department of Public Health Sciences, University of California, Davis, California, United States; 7 Ministry of Health Advisor, Maputo, Mozambique; 8 CUNY School of Public Health at Hunter College, Epidemiology and Biostatistics Program, New York, United States

**Keywords:** pre-ART, Mozambique, loss to care, retention, pregnancy, operations research, PEPFAR, ART eligibility

## Abstract

**Introduction:**

Retention in HIV care prior to ART initiation is generally felt to be suboptimal, but has not been well-characterized.

**Methods:**

We examined data on 37,352 adult pre-ART patients (ART ineligible or unknown eligibility) who enrolled in care during 2005–2008 with >1 clinical visit at 23 clinics in Mozambique. We defined loss to clinic (LTC) as >12 months since the last visit among those not known to have died/transferred. Cox proportional-hazards models were used to examine factors associated with LTC, accounting for clustering within sites.

**Results:**

Of 37,352 pre-ART patients, 61% had a CD4 count within three months of enrolment (median CD4: 452, IQR: 345–611). 17,598 (47.1%) were ART ineligible and 19,754 (52.9%) were of unknown eligibility status at enrolment because of missing information on CD4 count and/or WHO stage. Kaplan-Meier estimates for LTC at 12 months were 41% (95% CI: 40.2–41.8) and 48% (95% CI: 47.2–48.8), respectively. Factors associated with LTC among ART ineligible patients included male sex (AHR_men_vs_non-pregnant women_: 1.5; 95% CI: 1.4–1.6) and being pregnant at enrolment (AHR_pregnant_vs_non-pregnant women_: 1.3; 95% CI: 1.1–1.5). Older age, more education, higher weight and more advanced WHO stage at enrolment were independently associated with lower risks of LTC. Similar findings were observed among patients whose ART eligibility status was unknown at enrolment.

**Conclusions:**

Substantial LTC occurred prior to ART initiation among patients not yet known to be eligible for ART, including nearly half of patients without documented ART eligibility assessment. Interventions are needed to target pre-ART patients who may be at higher risk for LTC, including pregnant women and patients with less advanced HIV disease.

## Introduction

While the rapid expansion of access to antiretroviral therapy (ART) to nearly 4 million patients in sub-Saharan Africa has been a major accomplishment of HIV care and treatment programs [1], loss to clinic (LTC) of patients enrolled in HIV care is a persistent challenge [2]. Prior studies attribute large numbers of LTC of ART patients to early mortality [3–7], but patients who are not yet eligible for ART are less likely to have died [8]. However, they remain at risk of late ART initiation [9, 10] and, consequently, increased risk of early mortality after starting ART [11–16]. Furthermore, pre-ART patients who are neither engaged in care nor on ART are more likely to transmit HIV to sex partners than those who engage in care and initiate early ART [17].

Growing recognition of the benefits of timely treatment and the consequences of losing pre-ART eligible patients is leading to increased awareness of the importance of regular CD4 and clinical monitoring before treatment eligibility [18, 19]. The limited data available suggest that the losses of pre-ART eligible patients are significant [8, 12]. A systematic literature review of patient retention in sub-Saharan Africa estimated that fewer than one-third of patients remain continuously in care between testing HIV-positive and starting ART; specifically, a median of 45% (range 5–58%) of patients were lost to care after enrolment. However, the quality and heterogeneity of the studies limit the interpretation of these results [20].

With 11.5% of adults 15–49 years old living with HIV in 2009, Mozambique has one of the highest HIV seroprevalence rates in sub-Saharan Africa and has rapidly scaled up HIV testing and ART availability in recent years [21]. We used routine data from 23 clinics from the national program to determine the proportion of ART-ineligible patients who were LTC. We also examined LTC among patients without documentation of ART eligibility because they make up a substantial portion of the patient population and they may have been deemed to be ART ineligible on initial clinical assessment without confirmation by CD4 count or WHO stage. We identified factors associated with LTC and described outcomes for patients who were not LTC.

## Methods

### Ethics statement

The study utilized data that are routinely collected by sites for service delivery and anonymized for analysis. This study is part of the Identifying Optimal Models of HIV Care and Treatment in Mozambique Collaboration, which was approved by the Mozambican National Ethics Committee, the Columbia University Medical Center IRB, the US Centers for Disease Control and Prevention, and PEPFAR's Office of the Global AIDS Coordinator.

### Study design and study population

We used routinely collected service delivery data on HIV-infected adult patients (≥15 years old) enrolled at 23 free HIV care and treatment clinics across Mozambique that were part of the Mozambican National ART Program that were receiving PEPFAR support via the ICAP-Columbia University. We conducted a retrospective cohort study of adults enrolled in care between January 2005 and December 2008. We reviewed patient outcomes through December 2009, allowing for at least one year of follow-up for all patients.

Patients were included in the study if they were not on ART or *not known to be eligible* for ART at enrolment. ART eligibility throughout the study period was defined as: 1) WHO stage IV disease, 2) WHO stage III and CD4 count <350 cell/µL, or 3) CD4 count <200 cells/µL irrespective of WHO stage. For baseline patient characteristics (i.e. weight, CD4 count, WHO stage), we used any documented measure obtained within three months of enrolment.

We divided the study population into two groups: 1) ART ineligible and 2) ART eligibility unknown if either CD4 count or WHO stage were missing within three months of enrolment. We analyzed data from patients who were ART ineligible or eligibility was unknown and who returned for at least one follow-up visit. We excluded those who presented for only one visit because patients are not considered enrolled into care until a physician has reviewed their file, which often did not occur until a second follow-up visit. We also excluded those who were known to have transferred care to another facility and those who were documented as dead after one visit.

### Data and measures

National guidelines during the study period recommended that HIV patients undergo a comprehensive medical history assessment with a physical examination at their first clinic visit. Due to high demands on CD4 machines and lack of sample storage (cold chain) at many clinics, patients were scheduled to return to clinic on a subsequent day to have a CD4 count drawn to assess ART eligibility when specimens could be processed on the same day. For patients not ART eligible, the guidelines specified semi-annual visits with clinical and CD4 count monitoring for ART eligibility.

Patient information collected during each visit was documented on national patient forms that were entered daily by trained data clerks into a Microsoft Access electronic database that was developed by ICAP-Mozambique and deployed at all 23 sites. Each clinic contributed patient-level data for the entire study period. Data quality assessments (reviewing completeness and accuracy of the data) were performed every six months, with feedback to clinic staff on areas needing improvement.

LTC was defined as having occurred in a patient when 12 or more months elapsed since the last documented clinic visit. Patients were censored from the analysis if they started ART or met eligibility criteria for starting ART. Those known to have died or transferred to another facility were also censored.

The LTC event date for a lost patient was defined as 90 days after the last documented visit date. We calculated the number of days in care before LTC (time to LTC) as the difference between enrolment and the LTC event dates.

### Statistical analysis

We compared characteristics of patients who were ART ineligible with those whose ART eligibility status was unknown at enrolment, using the Chi-square test for categorical data and student *t*-test for continuous variables. We used Kaplan-Meier estimates to compute the proportion LTC at 6 and 12 months after enrolment. We used Cox proportional-hazards models, accounting for clustering at site level, to identify factors associated with LTC separately for the ART ineligible and ART eligibility unknown populations in bivariate and multivariate analyses.

Sex, age, marital status, number of children, socio-economic status, education, weight, CD4 count and WHO stage at enrolment were examined in the univariate models. We created a scale of socio-economic status (zero to two points), assigning one point each for having electricity and refrigeration. Models were adjusted for year of enrolment; missing data for each factor was modelled as a separate category. All variables from the univariate models were included in the final multivariable model. Associations were examined at a *p*<0.05 significance level (2-sided test). Multiple imputations with sensitivity analyses were conducted to assess the potential impact of missing data. Data were analyzed using SAS software 9.2 (SAS Institute, Cary, NC).

## Results

### Study population

Overall, 57,880 pre-ART patients not yet known to be clinically eligible for ART enrolled in HIV care at 23 clinics between January 1, 2005 and December 31, 2008. The population was divided into ART ineligible (17,775; 30.7%) and ART eligibility unknown (40,105; 69.3%). More than one-third of the population (19,954) had no follow-up after the first clinic visit and was excluded from further analysis.

The final study population was 37,352 pre-ART patients (17,598 ART ineligible and 19,754 eligibility unknown). Table 1 shows that the study population was 70.7% women and had a median age of 31.0 years. Approximately 8.5% of ART ineligible women and 7.0% of women with unknown ART eligibility were pregnant at enrolment. More than half of ART ineligible patients (54%) were married or in union and had completed primary school. Nearly three-quarters of the study population was documented as living without electricity or a freezer.

**Table 1:** Baseline characteristics of pre-ART adults enrolled in care with at least one follow-up visit

	Total Pre-ART		ART ineligible		ART eligibility unknown	
			
	N=37,352		N=17,598		N=19,754	
			
	n	Percentage	n	Percentage	n	Percentage
Sex						
Male	10,946	29.3	5,084	28.9	5862	29.7
Female (not pregnant at enrolment)	23,526	63.0	11,013	62.6	12,513	63.3
Female (pregnant at enrolment)	2880	7.7	1501	8.5	1379	7.0
Age, mean in years (median, IQR)	33.3	31 (25–40)	33.2	31 (25–40)	33.4	31 (25–40)
Age, intervals						
15–25	9806	26.3	4666	26.5	5140	26.0
26–35	14,147	37.9	6659	37.8	7488	37.9
36–45	8215	22.0	3873	22.0	4342	22.0
46–55	3800	10.2	1761	10.0	2039	10.3
>55	1384	3.7	639	3.6	745	3.8
Marital status						
Married/in union	18,523	49.6	9550	54.3	8973	45.4
Single	11,143	29.8	5212	29.6	5931	30.0
Widowed	2544	6.8	1276	7.3	1268	6.4
Missing/other	5142	13.8	1560	8.9	3582	18.1
Number of children, mean (median, IQR)	2.1	2 (0–3)	2.3	2 (0–3)	2.0	1 (0–3)
n>1 child	25,725	68.9	12,954	73.6	12,771	64.7
Education						
None or did not finish primary school	2218	5.9	1286	7.3	932	4.7
Primary school	19,754	52.9	9495	54.0	10,250	51.9
Secondary school or higher	7631	20.4	4069	23.1	3562	18.0
Missing	7758	20.8	2748	15.6	5010	25.4
SES						
Lower (no electricity)	27,889	74.7	12,157	69.1	15,732	79.6
Low (electricity only)	3598	9.6	2148	12.2	1450	7.3
Higher (electricity and freezer)	5861	15.7	3291	18.7	2570	13.0
Missing	4	0.0	2	0	2	–
Year of enrolment						
2005	5850	15.7	1484	8.4	4366	22.1
2006	9000	24.1	4709	26.8	4291	21.7
2007	11,213	30.0	5675	32.3	5538	28.0
2008	11,289	30.2	5730	32.6	5559	28.1
Point of entry						
VCT	14,382	38.5	7897	44.9	6485	32.8
PMTCT	3115	8.3	1490	8.5	1625	8.2
TB/HIV	232	0.6	101	0.6	131	0.7
Inpatient	2184	5.9	1015	5.8	1169	5.9
Outpatient	3006	8.1	1721	9.8	1285	6.5
Other	2541	6.8	771	4.4	1770	9.0
Missing/unknown	4240	11.4	1132	6.4	3108	15.7
Referral from another testing facility	7652	20.5	3471	19.7	4181	21.2
Weight at enrolment (in Kg)*						
Mean (median, IQR)	56.7	55.5 (50–62)	57.4	56 (50–63)	55.7	55 (49–61)
Missing weight	11,532	30.9	2784	15.8	8748	44.3
WHO stage at enrolment*						
Stage I	10,871	29.1	7636	43.4	3253	16.4
Stage II	7883	21.1	5232	29.7	2651	13.4
Stage III	9495	25.4	4730	26.9	4765	24.1
Stage IV	*	*	*	*	*	*
Missing baseline WHO stage	9,103	24.4	*	*	9103	46.1
CD4 count at enrolment*						
≤200	*	*	*	*	*	*
>200 and ≤275	2852	7.6	1943	11.0	909	4.6
>275 and ≤350	3090	8.3	2150	12.2	940	4.8
>350 and ≤500	7633	20.4	6146	34.9	1487	7.5
>500	9248	24.8	7359	41.8	1889	9.6
Missing baseline CD4 count	14,529	38.9	*	*	14,529	73.6
Mean (median, IQR)	502.7	452 (345–611)	510.4	461 (357–619)	476.9	422 (306–588)

* Documented within three months of first clinic visit. The ART ineligible and ART eligibility unknown populations are significantly different (p<0.0001) for all characteristics except median age (p=0.05) and age intervals (p=0.68).

Between 2005 and 2006, the proportion of ART ineligible patients that enrolled in care increased from 25.4% (1484 of the 5850) to 52.3% (4709 of 9000) of pre-ART patients (ART ineligible or eligibility unknown); the proportion of ART ineligible patient enrolment stabilized in 2007 and 2008 at just over 50%. The enrolment of patients whose ART eligibility status was unknown was 74.6% (4366) of pre-ART patient enrolment in 2005 and 49.2% (5559) in 2008. The largest proportion of patients was referred into care from VCT; significant proportions were also referred from “other testing facilities” and PMTCT. Of note, point of entry data was missing for a high proportion of ART eligibility unknown patients (along with marital status, education and weight).

Within three months of enrolment, 61% of pre-ART patients had a CD4 count measurement with a median of 452 cells/µL (IQR: 345–611 cells/µL). Of those who were ART ineligible (N =17,598), 12,992 (73.8%) remained ART ineligible for the duration of follow-up (median follow-up time=213 days; IQR 113–472 days). The remainder of the ART ineligible patients either met criteria for ART eligibility (4.6%) or started ART (21.6%).

Of those whose ART eligibility status was unknown at enrolment (N=19,754), 2756 (14.0%) were eventually staged as ART ineligible and 1,169 (5.9%) were staged as ART eligible but not started on ART at a median of 207 days and 235 days after enrolment, respectively. An additional 4,711 (23.8%) were started on ART. The majority, 11,118 (56.3%), neither completed eligibility assessment nor started ART at the clinic where they initially enrolled before the end of observation in 2009.

### Loss to clinic of ART ineligible patients and patients with unknown ART eligibility

Nearly half of the study population (17,722; 47.4%) was LTC during the observation period. Of the 12,992 patients who remained ART ineligible, 7633 (58.8%) were LTC while 4,593 (35.4%) remained in care during the study period. On average, patients who were ART ineligible at enrolment had three clinic visits during the study follow-up period. The Kaplan-Meier estimate of LTC at 12 months for ART ineligible patients was 41% (95% CI: 40.2–41.8%) and those lost were LTC at a median 137 days after enrolment. The median CD4 count last documented for LTC patients was 482 cells/µL [IQR: 368–648]. The distribution of last known WHO stages among LTC patients was 47% stage I, 28% stage II and 25% stage III.

Patients whose ART eligibility status was unknown at enrolment and were LTC were lost at a median 124 days after enrolment. Most of those who were LTC were lost after a second clinic visit (81.6%). The Kaplan-Meier estimate of LTC of patients whose ART eligibility status was unknown at enrolment was 48% (95% CI: 47.2–48.8%) at 12 months after enrolment. Of those who had a CD4 count before LTC (24.1%), the median CD4 count was 447 cells/µL [IQR: 329–621]. The last documented WHO stages before LTC showed a distribution of 34% stage I, 26% stage II and 40% stage III.

Among those patients who were known to be ART ineligible at enrolment, the proportion of patients that remained in care at the clinic through ART initiation during the four years of observation was 21.6%; an additional 30.7% were alive and in care at the clinic, but had not initiated ART by the end of the study period. The median CD4 count at ART initiation was 226 cells/µL (IQR: 176–311). Of those whose ART eligibility status was unknown at enrolment, 23.9% eventually started ART, with a median CD4 count at ART initiation of 196 cells/µL (IQR: 125–277).

### Factors associated with loss to clinic

Table 2 shows the results of crude and multivariable Cox proportional hazards models with LTC as the outcome. Among the ART ineligible patients, male sex was associated with a significantly higher risk of LTC (adjusted hazards ratio [AHR]_men_vs_non-pregnant women_: 1.48; 95% CI: 1.39–1.57). Among women, being pregnant at enrolment was associated with a higher risk of LTC (AHR_pregnant_vs_non-pregnant women_: 1.26; 95% CI: 1.08–1.46). Compared to the youngest age group of 15–25 year olds, older age was associated with a lower likelihood of LTC (AHR: 0.55; 95% CI: 0.48–0.64 for >45 years). More education (AHR_primary_vs_none_: 0.75; 95% CI: 0.61–0.92) and higher weight (AHR_>__56 kg_vs≤56 kg_: 0.86; 95% CI: 0.80–0.93) were also associated with lower risks of LTC. Compared to WHO stage I, more advanced stages of clinical disease were associated with lower hazards of LTC (AHR: 0.85; 95% CI: 0.77–0.95 for WHO stage II).

**Table 2:** Multivariable analysis of factors associated with LTC for ART ineligible population

	ART ineligible (N=17,598)				ART eligibility unknown (N=19,754)			
		
Variable*	Crude HR	95% CI	AHR	95% CI	Crude HR	95% CI	AHR	95% CI
Sex								
Non-pregnant female	1		1		1		1	
Male (male vs. non-pregnant female)	1.25	1.17–1.33	1.48	1.39–1.57	1.24	1.18–1.30	1.42	1.33–1.51
Pregnancy (pregnant vs. non-pregnant female)	1.58	1.35–1.84	1.26	1.08–1.46	1.56	1.22–2.00	1.19	1.00–1.43
Age								
15–25	1		1		1		1	
26–35	0.73	0.67–0.79	0.75	0.69–0.82	0.74	0.64–0.84	0.79	0.72–0.86
36–45	0.58	0.52–0.65	0.60	0.55–0.67	0.66	0.57–0.75	0.73	0.69–0.79
>45	0.57	0.49–0.65	0.55	0.48–0.64	0.61	0.51–0.72	0.65	0.59–0.72
Marital status								
Married/in union	1		1		1		1	
Single	1.02	0.94–1.11	1.00	0.92–1.10	1.02	0.93–1.11	1.00	0.91–1.11
Widowed	0.76	0.62–0.94	0.93	0.78–1.11	0.76	0.60–0.96	0.90	0.76–1.08
Missing/other	1.10	0.92–1.31	0.99	0.78–1.26	1.06	0.85–1.31	1.16	0.86–1.56
Number of children								
0	1		1		1		1	
1	0.94	0.87–1.03	0.99	0.91–1.08	0.94	0.80–1.10	0.94	0.79–1.02
2	0.82	0.74–0.90	0.91	0.82–1.02	0.87	0.75–1.00	0.87	0.76–0.99
3 or more	0.74	0.65–0.83	0.93	0.81–1.06	0.74	0.64–0.86	0.74	0.67–0.91
Education								
None or did not finish primary school	1		1		1		1	
Primary school	0.84	0.67–1.05	0.75	0.61–0.92	0.80	0.70–0.91	0.78	0.69–0.89
Secondary school or higher	0.78	0.61–1.00	0.66	0.53–0.81	0.66	0.58–0.74	0.60	0.53–0.67
Missing	0.96	0.72–1.29	0.91	0.69–1.21	0.72	0.55–0.95	0.80	0.64–1.00
SES								
Lower (no electricity)	1		1		1		1	
Low (electricity only)	0.94	0.82–1.08	0.98	0.86–1.11	1.12	0.96–1.30	1.10	0.99–1.21
Higher (electricity and freezer)	0.81	0.71–0.92	0.90	0.80–1.03	0.78	0.64–0.96	0.89	0.82–0.98
Weight at enrolment (in kg)**								
<or=56kg	1		1		1		1	
>56 kg	0.85	0.77–0.93	0.86	0.80–0.93	0.84	0.77–0.91	0.81	0.76–0.86
Missing weight	0.84	0.59–1.20	0.95	0.70–1.29	0.76	0.50–1.17	0.84	0.70–1.01
WHO stage at enrolment***								
Stage I	1		1		1		1	
Stage II	0.81	0.73–0.89	0.85	0.77–0.95	0.92	0.80–1.05	0.93	0.85–1.01
Stage III	0.85	0.76–0.97	0.87	0.77–0.99	0.85	0.76–0.96	0.94	0.81–1.10
Stage IV	*	*	*	*	*	*	*	*
Missing baseline WHO stage	*	*	*	*	1.12	0.63–2.01	1.00	0.77–1.31
CD4 count at enrolment***								
>200 and ≤275	1		1		1		1	
>275 and≤350	0.98	0.90–1.07	0.97	0.89–1.06	0.96	0.83–1.11	0.95	0.82–1.11
>350 and≤500	0.97	0.89–1.05	0.95	0.87–1.03	0.98	0.87–1.11	0.96	0.84–1.09
>500	1.12	1.01–1.25	1.07	0.97–1.18	1.10	0.92–1.32	1.04	0.88–1.23
Missing baseline CD4 count	*	*	*	*	0.65	0.37–1.14	0.60	0.47–0.78

* Multivariable model was adjusted for year of enrolment.

** Median of 55 kg used for ART eligibility unknown population; weight documented within three months of first clinic visit.

*** Documented within three months of first clinic visit.

Similar findings were observed among patients whose ART eligibility status was unknown at enrolment (Table 2). Male sex was associated with a higher risk of LTC (AHR_men_vs_non-pregnant women_: 1.42; 95% CI: 1.33–1.51). Pregnancy was again associated with a higher risk of LTC, though at a borderline significant level (AHR_pregnant_vs_non-pregnant women_: 1.19; 95% CI: 1.00–1.43). Older age, higher education levels and more children were associated with lower risks of LTC. Higher weight (AHR_>__56 kg_vs_≤56 kg_: 0.81; 95% CI: 0.76–0.86) was also associated with a significantly lower risk of LTC. Access to both a freezer and electricity were associated with a significantly lower risk of LTC (AHR_both_vs_none_: 0.89; 95% CI: 0.82–0.98).

CD4 counts at enrolment were not significantly associated with LTC for either group of patients. Missing CD4 count at enrolment was associated with a lower risk of LTC among those for whom ART eligibility was unknown. Results of multiple imputation of missing CD4 values were highly consistent in terms of the direction and magnitude of association; *p*-values in sensitivity analyses were not meaningfully different from those presented in Table 2.

### Subanalyses of pregnant women

**Figure 1:**
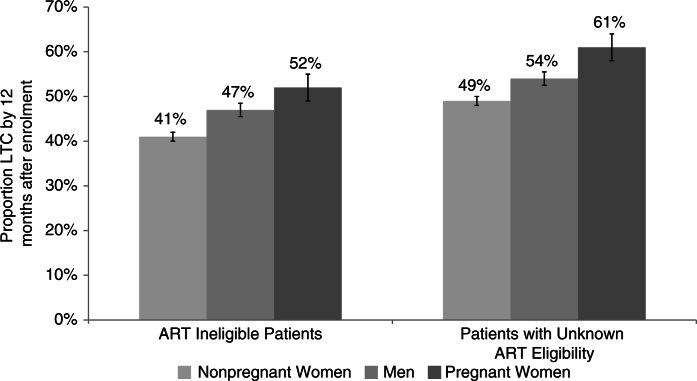
LTC of ART ineligible patients and patients with unknown ART eligibility 12 months after enrolment.

There were 2880 women who were documented as being pregnant that enrolled in pre-ART care between January 2005 and December 2008. Figure 1 shows that pregnant women were more likely to be LTC than men and non-pregnant women. Approximately 57% of all pre-ART pregnant women were LTC at a median of 120 days after enrolment. On average, pregnant women were LTC sooner than other women and men; LTC appears to peak in the peri-partum period (n=1442; median time of LTC: 42 weeks since last menstrual period [LMP]; IQR: 36–51 weeks since LMP).

### ART eligibility staging and loss to clinic by site

At any of the 23 sites, the proportion of pre-ART patients whose ART eligibility was documented within three months of enrolment ranged from 7.1 to 78.6%. The proportion of pre-ART patients who were LTC during the study period ranged from 29.5 to 73.7%. The number of pre-ART patients who enrolled in HIV care between January 2005 and December 2008 at each site (clinic patient load) did not correlate with the proportion of patients assessed for ART eligibility or the proportion LTC from each site.

## Discussion

Our results document the high magnitude of LTC prior to ART eligibility and initiation across several sites and settings in Mozambique, with a 12-month LTC proportion of 41–48% among those with >1 clinical care visit. The proportion of 37,352 patients who were not yet known to be eligible for ART at enrolment and continued in care until ART initiation during the study period is 23%, with an additional 25% remaining in care at the site where they initially enrolled until the end of follow-up. The large portion of patients who were lost after just one clinic visit (19,783, or 34.5% of 57,880 pre-ART patients) further magnifies the problem of pre-ART attrition. These findings are relatively consistent with a recent systematic literature review that estimated fewer than one third of ART ineligible patients remain in continuous care until ART initiation [20].

This study adds to the evidence that expansion of HIV testing and counselling does not necessarily translate into timely care engagement and CD4 testing [9, 22–24]. Our study showed that 39% of patients not yet known to be ART eligible did not have a documented CD4 count within three months of their first visit. Providers’ perceptions of these patients as healthy as well as the lack of access to immediate CD4 testing may have led them to consider these patients as ART ineligible without checking CD4 counts or formally documenting disease stage, despite recommendations to do both.

The use of point-of-care CD4 count technology at the first visit may be one strategy for more rapid eligibility assessment [25] and may help reduce the substantial loss of patients we have seen after the first clinic visit. The opportunity for patients to provide a sample and receive a test result at the first visit might engage them in care by raising their HIV-related health literacy and awareness before logistical barriers such as lack of transportation, absence from work and fear of stigma compete with their need and desire to receive ongoing care [26–30]. Researchers should continue to evaluate point-of-care testing as a strategy to improve patient retention in pre-ART care [31–34].

Nevertheless, the substantial loss of patients even after ART eligibility assessment suggests that more than CD4 testing and clinical staging is necessary to retain ART ineligible patients in care. Current guidelines do not offer therapeutic interventions to asymptomatic patients with high CD4 counts (i.e. >350 cells/µL), leaving patients without concrete reasons such as medications to return for visits. One recent study showed higher retention among pre-ART patients who received prophylaxis for opportunistic infections (OI), such as cotrimoxazole or isoniazid, compared with those who did not [35]. Although this strategy presents risks of medication side effects, expanding cotrimoxazole eligibility to all pre-ART patients may improve pre-ART retention rates, and requires more investigation. We were unable to assess the role of OI prophylaxis in our study due to incomplete data. As HIV treatment guidelines expand and support grows for the “test and treat strategy” [36], expanded or a universal offering of ART may be implemented in the future, but prevention of LTC requires more immediate and sustainable interventions.

With women of reproductive age representing a growing percentage of people enrolling in HIV care in Mozambique [37], the higher risk of LTC observed among pregnant women is particularly concerning. PMTCT programs refer pregnant women into HIV care, but more support is needed to link, engage and retain pregnant women once they enroll in care, especially pregnant women in pre-ART care, as they need to initiate and maintain treatment with ARVs both for their own health and to prevent vertical transmission. Our study showed that LTC among women who were pregnant at enrolment peaked in the peri-partum period, perhaps as a consequence of increased domestic demands in caring for the newborn. Other studies also show poor retention of peri-partum women, including one study of women who started ART while pregnant and found higher rates of non-adherence to ART after delivery [38]. Anticipating the needs of pregnant women and providing them with outreach care and support may be an effective means of keeping them engaged in care, especially during the vulnerable peri-partum period [39].

This study is responsive to the urgent call for researchers to closely examine the problem of LTC amongst patients not yet eligible for ART [8, 20, 30, 40]. While others have studied isolated phases of pre-ART care [8, 26, 30, 41–43], this study fills an important gap in the literature by documenting the proportion of patients who enrolled in care before ART eligibility and remained in care at until treatment initiation. Furthermore, our data suggest that patients who enrol and remain in care prior to ART eligibility eventually start ART at median CD4 counts more in line with recommendations by national guidelines, as compared with the majority of ART patients that report medians much lower than the threshold of 200 cells/µL [4, 9]. This suggests that it is possible at least for some patients to remain relatively continuously in pre-ART care over longer periods such that appropriate monitoring can occur, with treatment initiated in a relatively timely fashion.

Our study has several findings and associated limitations related to missing data. Poor documentation, incomplete data entry, limited access to CD4 measurement or a combination of these factors are likely reasons for substantial missing information on ART eligibility at enrolment and soon thereafter. The substantial portion of patients missing information on ART eligibility criteria suggests that problems in patient monitoring and data tracking are system-wide and are adversely affecting rates of LTC. However, we could also not quantify the extent to which information may have been missing as a result of patients becoming LTC.

We could not report outcomes for those who are LTC, including whether patients lost to clinic are in fact lost to or disengaged from HIV care altogether; undocumented transfers may account for a large proportion of patients LTC, especially in the context of rapid scale-up and decentralization of HIV programs during the study period. Similarly, the proportion of lost patients who died is unknown but likely small for ART ineligible patients, although high rates of maternal mortality in Saharan Africa, where 1 in 31 women die during childbirth (ranging from 470 to 930 deaths per 100,000 live births), may contribute [44], especially among those LTC during the peri-partum period. As a result, the proportion of patients who actually disengaged from HIV care is most likely less than the proportion our study reports as LTC. Research is needed to characterize the outcomes among these patients, as well as the reasons for dropping out of care, as has been done for ART patients [2, 45].

Our findings on pregnant pre-ART women have important implications for program implementers, but they should be interpreted with caution. Pregnancy status and gestational age were derived from patients’ verbal reports or documents from other clinical services and were not verified by staff entering the data. It is unclear how many women were missing pregnancy status because they were never asked or the information was never documented or entered; further, only half of the women documented as pregnant also had documented gestational age at enrolment. Finally, we studied pregnancy only at enrolment and could not assess whether becoming pregnant after enrolment is also a risk factor for LTC, which would further raise our concern that pregnancy may be an especially vulnerable period that should be the focus of targeted interventions.

## Conclusions

LTC among patients not yet eligible for ART or for whom ART eligibility has not been assessed or documented is a major barrier with the potential to undermine the success of HIV care and treatment programs in sub-Saharan Africa. While LTC of ART ineligible patients does not usually portend immediate death, it perpetuates the pattern of late ART initiation [9, 10, 46] and contributes to the high mortality observed during the first year on ART. Furthermore, losing patients who present for care is a missed opportunity to prevent onward HIV transmission. Where resources allow, we must better understand how to target and intervene with patients prior to ART eligibility, especially men, pregnant women and others who are at higher risk of LTC. Improved patient tracking systems and more research on predictors and outcomes for pre-ART patients will help shape the development of effective interventions and programmatic activities, especially as ART eligibility criteria are expanding or are slated for expansion in many resource-limited settings. Future studies should investigate reasons for missing ART eligibility monitoring data, ascertain outcomes among those LTC, and test interventions to retain pre-ART patients.

## Competing interests

The authors have no competing interests to declare.

## Authors' contributions

All investigators contributed to concept of the study. RP analyzed data and wrote the manuscript. ML and DN supervised data analysis and revised the manuscript. HB conducted statistical analysis and edited the manuscript. BS participated in study design and edited the manuscript. All other authors critically read and approved the final manuscript.
